# Caffeine enhances upper body strength in resistance-trained women

**DOI:** 10.1186/1550-2783-7-18

**Published:** 2010-05-14

**Authors:** Erica Goldstein, Patrick L Jacobs, Michael Whitehurst, Tina Penhollow, Jose Antonio

**Affiliations:** 1 Department of Exercise Science and Health Promotion, Florida Atlantic University, Boca Raton, FL 33431, USA; 2 Nova Southeastern University, Fort Lauderdale-Davie, FL 33314, USA

## Abstract

**Background:**

Research has indicated that low-to-moderate dosages of caffeine supplementation are ergogenic for sustained endurance efforts as well as high-intensity exercise. The effects of caffeine supplementation on strength-power performance are equivocal, with some studies indicating a benefit and others demonstrating no change in performance. The majority of research that has examined the effects of caffeine supplementation on strength-power performance has been carried out in both trained and untrained men. Therefore, the purpose of this study was to determine the acute effects of caffeine supplementation on strength and muscular endurance in resistance-trained women.

**Methods:**

In a randomized manner, 15 women consumed caffeine (6 mg/kg) or placebo (PL) seven days apart. Sixty min following supplementation, participants performed a one-repetition maximum (1RM) barbell bench press test and repetitions to failure at 60% of 1RM. Heart rate (HR) and blood pressure (BP) were assessed at rest, 60 minutes post-consumption, and immediately following completion of repetitions to failure.

**Results:**

Repeated measures ANOVA indicated a significantly greater bench press maximum with caffeine (p ≤ 0.05) (52.9 ± 11.1 kg vs. 52.1 ± 11.7 kg) with no significant differences between conditions in 60% 1RM repetitions (p = 0.81). Systolic blood pressure was significantly greater post-exercise, with caffeine (p < 0.05) (116.8 ± 5.3 mmHg vs. 112.9 ± 4.9 mmHg).

**Conclusions:**

These findings indicate a moderate dose of caffeine may be sufficient for enhancing strength performance in resistance-trained women.

## Background

Caffeine is naturally derived from ordinary food items such as tea leaves, cocoa, coffee beans, and chocolate [1,2] and commonly consumed in the form of coffee, tea, and carbonated beverages[1,3]. Various physiological mechanisms associated with the ergogenic effects of caffeine have been described in the literature. It has been suggested that caffeine is an adenosine antagonist [4,5] and the primary mode of action may be on the central nervous system [6]. Other studies have suggested that caffeine may also have the ability to alter substrate utilization by acting to increase fat oxidation and, thus, spare glycogen utilization [7,8]. In addition, studies have also indicated enhanced secretion of β-endorphins [9] during exercise with a subsequent decrease in pain perception [10], as well as an enhanced thermogenic response [11] and alteration of neuromuscular function and/or skeletal muscular contraction [12,13].

The ergogenic properties of caffeine have been extensively studied and research has indicated that low-to-moderate (~3-6 mg/kg) dosages of caffeine supplementation are ergogenic for sustained endurance efforts [7,14-17] as well as high-intensity exercise [18-20]. The effects of caffeine supplementation on strength-power performance are equivocal, with some studies indicating a benefit [18,21] and others demonstrating no significant change in performance [22,23]. In fact, a number of investigations have indicated that in trained males, a low-to-moderate dose of caffeine (~2-6 mg/kg) was effective for significantly enhancing upper body strength performance [18,21]. However, other studies have suggested that with similar doses of caffeine no significant changes in upper body strength were apparent [22,23]. The difference in outcomes between these studies could be the result of a range of intensity within the separate protocols and levels of habituation to caffeine within subjects.

Investigations that have examined these same dynamics in women are scarce and vary in design and level of condition of the participants studied. Ahrens and colleagues reported results for two different investigations [24,25] that examined the effects of low-to-moderate (3-6 mg/kg) dosages of caffeine on moderate aerobic exercise in untrained women. In the first study [24] results indicated a significant increase in energy expenditure, but no effect on measures of heart rate (HR), respiratory exchange ratio, or rating of perceived exertion (RPE). Results of the second investigation [25] indicated no significant increase in performance for caffeine supplementation and muscular endurance. Finally, Anderson et al. [26] reported a significant improvement in time among trained oarswomen for a 2,000 m row, following what is considered to be a high dose of caffeine (9 mg/kg).

As suggested, the design and population of women studied in relation to caffeine supplementation is varied. In addition, there are no investigations in the available literature that report any outcomes related specifically to resistance-trained females. Therefore, the purpose of this study was to determine the acute effects of caffeine supplementation on strength and muscular endurance in resistance-trained women.

## Methods

### Research Participants

Fifteen resistance-trained females volunteered to serve as research participants for this investigation. Inclusion criteria stipulated that all subjects were between the ages of 18-45 and had participated in resistance training activities at least 3-5 days per week for the 6 month period immediately prior to enrollment in this study. Other inclusionary criteria included the ability to bench press 70% of individual body weight. All testing procedures were verbally explained in detail and subjects provided written informed consent prior to participation, in accordance with the guidelines established by the Institutional Review Board at a southeastern university.

### Study Protocol

A double-blind, placebo-controlled, cross-over design was utilized in this investigation. Research participants were asked to attend three laboratory sessions. The first session was for familiarization, followed by two testing trials seven days apart using the same testing protocol. Either caffeine at a dose of 6 mg/kg or placebo (PL) was administered orally 60 minutes prior to testing, in randomized order (See Supplementation Protocol Section).

Research participants were directed to continue the same general lifestyle patterns of exercise and nutrition intake during each seven-day period prior to the two exercise testing sessions. To verify the consistency of diet, the subjects were directed to complete a 3-day dietary recall (two week days and one weekend day) for each week prior to testing. The dietary intake data were analyzed using ESHA Food Processor SQL dietary analysis software (ESHA Research, Salem, OR).

All research participants completed one familiarization session prior to participating in the two testing trials. During the familiarization session, the participants were instructed on proper technique and mechanics of the bench press exercise, according to the standard methods defined by Baechle and Earle [27] and the National Strength and Conditioning Association. Additionally, participants performed a series of lifts to determine their ability to bench press 70% of individual body weight.

On test days, participants were asked to report to the testing laboratory in the morning after a 12-hour period without food. Subjects were asked to refrain from participating in vigorous activity and to avoid the consumption of caffeinated food and beverages in the 24-hour period prior to testing.

### Supplementation Protocol

The two exercise trials were performed under two conditions, one with caffeine and one without. The study supplementation consisted of 6 mg/kg of caffeine provided in powder form (Scivation/Primaforce, Graham, North Carolina), which was mixed with 16.9 ounces of commercially available flavored Propel^® ^Fitness Water. Each 16.9-ounce serving of Propel^® ^Fitness Water is 20 calories and five grams of carbohydrates. The placebo was the single 16.9-ounce serving of flavored Propel Fitness Water. The supplement assignments were blinded to both the research participants and the study investigators. Participants ingested the respective 6 mg/kg of caffeine or PL approximately 60 minutes prior to testing.

### Testing Protocol

The assessment protocol consisted of testing to determine one repetition maximum (1RM) and repetitions to failure (RF) at 60% on a standardized barbell bench press. One-repetition maximum was determined in three to six sets with 2-minute rest intervals between sets [22]. One-repetition maximum was estimated using data from the familiarization trial and the Mayhew regression equation. The participant completed a warm-up by performing 12-15 repetitions at 50% of anticipated maximum. The participant then performed five repetitions starting at 60% of anticipated 1RM. If the participant was successful lifting the weight five times, resistance was increased by 15% with three required lifts. The weight was then increased to 90% of estimated 1RM, with one required lift. If the participant was successful, the weight was then increased to 100% of estimated 1RM, or until the participant failed to complete a lift. The participant then rested for five minutes before completing the test for muscular endurance. The participant completed as many bench press repetitions as possible at 60% 1RM to assess muscular endurance [22].

Within five seconds of completing the final repetition, HR, blood pressure (BP), and RPE were recorded. Heart rate was measured using a Polar HR monitor system, and BP was recorded by manual sphygmomanometry. All measurements were performed by the same technician. Total weight lifted was determined as repetitions × weight.

### Statistics

All outcome measures were statistically examined to determine whether there were significant differences between conditions (Caffeine, PL) using one-way ANOVA procedures with repeated measures. In all cases, a p-value of less than 0.05 was accepted to determine statistical significance. All data analyses were performed using SPSS, Version 16.

## Results

Fifteen resistance-trained women completed both exercise trials. The study participants were aged 24.6 ± 6.9 years with a mean body mass of 63.6 ± 8.3 kg and stature of 166.2 ± 9.0 cm. The 15 study participants were confirmed to satisfy the inclusion criteria, and demonstrated the ability to perform bench press repetitions to failure at a resistance that was equivalent to 70% of individual body weight.

### Dietary log data

Macronutrient intake values for both study conditions are presented in Table 1. Dietary intake data for protein (g), carbohydrates (g), and fats (g) as well as total calories were analyzed to determine daily averages, which were compared between study conditions. Analysis indicated that there were no significant differences in these nutrient values for the three-day periods preceding each of the two exercise trials.

**Table 1:** Nutrient consumption three days prior to each experimental protocol (means ± SD).

	Placebo	Caffeine
Total energy (kcal)	2160 ± 1008	2083 ± 1095
Protein (g)	103 ± 46	102 ± 39
Carbohydrate (g)	252 ± 144	256 ± 186
Fat (g)	145 ± 274	117 ± 181

### Strength and Muscular Endurance

Analysis indicated a significantly greater bench press maximum with caffeine (p < 0.05) (52.9 ± 11.1 kg vs. 52.1 ± 11.7 kg). No significant differences were observed between conditions for 60% 1RM repetitions (p = 0.81) (Table 2). Caffeine consumption within subjects ranged from 0-416 mg per day. Eight subjects consumed ≤ 250 mg per day and seven consumed ≥ 250 mg per day.

**Table 2:** Muscle strength and endurance data (means ± SD).

	Placebo	Caffeine
Bench Press		
1RM (kg)	52.1 ± 11.7	52.9 ± 11.1*
60% 1RM	23.0 ± 7.1	23.1 ± 6.2

* Indicates significant difference between conditions, *p *< 0.05.

### Heart Rate and Blood Pressure

Heart rate and BP were recorded at rest, 60 min following ingestion of the supplement (Caffeine, PL), as well as immediately post-exercise (see Table 3). No differences were observed for HR at any of the three time points. There was no difference between conditions for diastolic blood pressure (DBP) either at rest, 60 min post-consumption, or immediately following exercise. There were no differences between conditions for systolic blood pressure (SBP) either at rest or 60 min following supplementation; however, SBP was significantly greater immediately following exercise with caffeine (p < 0.05) (116.8 ± 5.3 mmHg vs. 112.9 ± 4.9 mmHg).

**Table 3:** Cardiovascular Response data (means ± SD).

	Placebo	Caffeine
Heart rate (bpm)		
Rest	68.3 ± 10.3	68.5 ± 13.3
60-min post supplementation	67.3 ± 10.2	70.0 ± 10.4
Immediately post exercise	90.0 ± 14.0	94.0 ± 16.0
Diastolic blood pressure (mmHg)		
Rest	63.3 ± 5.0	65.0 ± 6.5
60-min post supplementation	63.0 ± 4.4	64.4 ± 5.3
Immediately post exercise	63.0 ± 4.5	64.3 ± 5.2
Systolic blood pressure (mmHg)		
Rest	109.4 ± 5.5	110.3 ± 5.2
60-min post supplementation	111.6 ± 6.8	111.0 ± 5.6
Immediately post exercise	112.9 ± 4.9	116.8 ± 5.3*

* Indicates significant difference between conditions, *p *< 0.05.

## Discussion

The major finding of this study is that acute caffeine supplementation appears to be effective for enhancing strength performance in resistance-trained women, as demonstrated by a significant increase in bench press 1RM. Caffeine supplementation at 6 mg/kg had no statistically significant effect on muscular endurance, HR, or DBP. Caffeine consumption did result in a 4 mmHg increase in SBP immediately following exercise testing, which included determination of 1RM, a 5-min rest interval, and RF at 60% of individual 1RM.

These results are in disagreement with Astorino et al. [22], as the authors of that investigation reported no significant increase in upper body strength in resistance-trained males after consuming 6 mg/kg of caffeine. However, the outcomes of research investigations that have examined the effects of low-to-moderate dosages of caffeine on strength-power performance have been somewhat inconsistent. Accordingly, no other studies have specifically examined the effects of caffeine supplementation on strength or muscular endurance in resistance-trained women. Recently, Woolf et al. [18] demonstrated that a moderate dose of caffeine (5 mg/kg) was effective for enhancing performance for the chest press and peak power on the Wingate. Participants in that study [18] were conditioned male athletes, and the results are similar to those of Beck and colleagues [21], who reported a significant increase in upper body strength following a low dose (2.1-3.0 mg/kg) of caffeine in resistance-trained males.

In contrast, a different group of authors found no increase in strength, for either the bench press or front latissimus dorsi pull down, following ingestion of either caffeine at an absolute dose of 300 mg, or the combination of caffeine plus ephedra (60 mg) [28]. In addition, a different study published by Beck et al. [29] reported no change in performance for untrained males, who received the same dose of caffeine 60 min prior to performing a 1RM test on the bench press. More recently, Woolf et al. [23] demonstrated that in non-habituated trained male athletes, caffeine supplementation (5 mg/kg) had no significant affect on bench press performance.

The dosage selected for the present study was based in part on the findings of Ahrens et al. [24]. In that study a moderate dose of caffeine (6 mg/kg) was effective for enhancing a metabolic response in untrained women. Ahrens et al. [24] also reported symptoms related to a high caffeine dose of 9 mg/kg. Women reported feelings of profuse sweating, body tremors, dizziness, and vomiting. The subjects in the present investigation reported a wide range in caffeine habituation as indicated by reported daily intake ranging from zero to 416 mg per day. Three of the 15 participants, who consumed either 0-41 mg per day, exhibited intense emotional responses, including an expressed inability to verbally communicate, focus, and/or remain still, as well as the feeling of wanting to cry. In addition, two of the three participants, who experienced an intense emotional response, demonstrated an improvement in performance during the muscular endurance portion of the protocol. In other words, these participants performed more repetitions to failure at 60% of individual 1RM.

Astorino et al. [22] reported symptoms that included restlessness, tremor, greater energy, and elevated HR. In addition, these feelings were augmented in those participants who consumed little caffeine on a daily basis. It is possible that caffeine consumption for some individuals will result in an enhancement in performance, second to feelings that present a loss of focus or emotional unrest. However, in other individuals the result may be in an increase performance without any presentable symptoms.

Therefore, the difference in outcomes between investigations that have examined the effect of caffeine supplementation and strength-power performance could be the result of a variation of intensity within the separate protocols, a difference in relative dosages of caffeine, and wide ranging levels of caffeine habituation. Participants in the Beck et al. [21] study consumed a low dose of caffeine and performed repetitions to failure at 80% of individual 1RM on the bench press. In contrast, the study design for the Astorino et al. [22] publication included repetitions to failure at 60% of individual 1RM on the bench press and a caffeine dosage of 6 mg/kg.

It is also possible that a magnitude of effect may exist, and it is greater for those individuals non-habituated to caffeine. Bell et al. [30] reported a positive effect on performance for participants classified as users (≥ 300 mg/d) and nonusers (≤ 50 mg/d) of caffeine. Individuals identified as nonusers exhibited a treatment effect at 6 hrs post consumption, which was not the case for users - this group only had a significant increase in endurance performance at 1 and 3 hours post consumption [30].

Other investigations have reported dissimilarity in performance between male and female athletes. Bruce et al. [20] used both a 6 and 9 mg/kg dose of caffeine when testing competitive oarsmen and women. In men [20], both dosages of caffeine were effective for enhancing time trial completion and average power output; however, the 9 mg/kg dose did not result in any further additional increases in performance. Results for the women [26] had an opposite effect: in a 2,000-m row, only the higher dose (9 mg/kg) resulted in a significant improvement in time. It is possible that a difference in response to caffeine supplementation exists between male and female athletes.

A second investigation published by Astorino et al. [31] examined cardiovascular responses to caffeine supplementation and resistance exercise in men. Systolic blood pressure was approximately 8-10 mmHg higher following caffeine ingestion and resistance exercise, as compared with placebo [31]. These results are comparable to the present investigation, where a significant increase in SBP occurred, but to a lesser extent of 4 mmHg.

Results published by Hartley et al. [32] also indicated an approximate 4 mmHg increase in BP following caffeine supplementation (3.3 mg/kg), but for both male and female subjects. Participants in the Hartley et al. [32] investigation did not perform exercise but instead performed mentally stressful tasks such as public speaking and reading aloud. The suggested mechanisms responsible for the increase in BP were different. Specifically, women responded to caffeine with an increase in cardiac output facilitated by an increase in stroke volume. Men, however, had no change in cardiac output but instead responded with an increase in peripheral resistance.

## Conclusion

In conclusion, the major finding of this study is that a 6 mg/kg dose of caffeine was effective for enhancing strength but not muscular endurance in resistance-trained women. This is a novel finding as it is the first investigation to examine caffeine supplementation among this population. These results are specific to trained women, and should not be generalized to both male and female athletes. It is also apparent that a limitation to this study is the small sample size. Recruiting resistance-trained women, specifically those with the ability to bench press 70% of individual body weight, was difficult. Specifically many recreationally trained women, who frequently participate in resistance training, underestimate the conditioning that is essential for a female to bench press a relatively high percentage of body weight. While inclusionary criteria of this study limited subjects to females, who possessed an acceptable level of upper body strength, it is recommended that future investigations examine the effects of a 6 mg/kg dose of caffeine on lower body strength and muscular endurance in resistance trained women.

In addition, it is also recommended that future investigations examine whether a lower dose of caffeine would stimulate a similar increase in strength performance, as indicated by results of this study, but without the intense emotional response that was experienced by some of the participants. Overall, results of this study indicate a moderate dose of caffeine prior to resistance-exercise may be beneficial for increasing upper body strength performance in resistance-trained women.

## Competing interests

The authors declare that they have no competing interests.

## Authors' contributions

All authors contributed to the study design and reviewed and contributed to the final manuscript. EG and PJ were responsible for data collection, statistical analysis, and manuscript preparation. All authors have read and approved the final manuscript.
